# Supplementary material for: Mid-infrared single-pixel imaging at the single-photon level
Source: Nat Commun. 2023 Feb 25;14:1073. doi: 10.1038/s41467-023-36815-3 (PMC9968282; doi:10.1038/s41467-023-36815-3)
Supplement: Supplementary file 1 — Supplementary Information [file 41467_2023_36815_MOESM1_ESM.pdf]

Mid-infrared single-pixel imaging at the single-photon level
# - Supplementary Information -

Yinqi Wang,[1] Kun Huang,[1,2,3,*] Jianan Fang,[1] Ming Yan,[1,2] E Wu,[1,2] and Heping Zeng[1,2,4,5,†]

[1]*State Key Laboratory of Precision Spectroscopy, East China Normal University, Shanghai 200062, China*

[2]*Chongqing Key Laboratory of Precision Optics, Chongqing Institute of East China Normal University, Chongqing, China*

[3]*Collaborative Innovation Center of Extreme Optics, Shanxi University, Taiyuan, Shanxi 030006, China*

[4]*Jinan Institute of Quantum Technology, Jinan, Shandong 250101, China*

[5]*Shanghai Research Center for Quantum Sciences, Shanghai 201315, China*

(Dated: January 25, 2023)

Supplementary Note 1: Theory of nonlinear structured detection

The nonlinear structured detection is the key to our realization of the mid-infrared (MIR) single-pixel imaging at the single-photon level. The involved nonlinear spatial modulation relies on an all-optical three-wave mixing process. To this end, the object image and pump patterns are steered into a nonlinear crystal to perform the sum-frequency generation (SFG). In our experiment, the field distribution of the MIR object image is formed by the product of the transverse profile of a Gaussian beam $E_1(x, y)$ and the transmission pattern of a mask target $E_O(x, y)$, which can be expressed as

$$E_s(x, y) = E_1(x, y) \times E_O(x, y) \propto e^{-(x^2+y^2)/w_1^2} \times E_O(x, y) \ , \tag{1}$$

where w_1 is the radius of the signal beam waist. In order to perform the SFG, the object image is scaled down into the crystal, leading to a field distribution of $E_s(-x/M_s, -y/M_s)$ at the crystal center, where M_s is the scaling factor for the relay optics. More rigorously, the spatial evolution along the longitudinal axis of the crystal should be taken into account. To this end, the Collins diffraction integral equation is used to investigate the propagation in the paraxial optical system, which reads as

$$\begin{aligned} U_2(x_2, y_2) &= \mathcal{F}[U_1(x_1, y_1), \mathcal{M}(d)] \\ &= \frac{1}{i\lambda B} \iint U_1(x_1, y_1) e^{-ikd - \frac{ik}{2B}[A(x_1^2+y_1^2)+D(x_2^2+y_2^2)-2(x_1x_2+y_1y_2)]} \, dx_1 dy_1 \ , \end{aligned} \tag{2}$$

where U_1 is the input field, U_2 is the propagation result, $\mathcal{M}$ is the ray transfer matrix (ABCD matrix), k is the optical wave number, and d is the axial optical distance between the two planes. Therefore, the spatial field distribution of the object image within the nonlinear crystal can be obtained by $E'_s(x, y, z) = \mathcal{F}[E_s(x, y), \mathcal{M}_s(z)]$, where $\mathcal{M}_s(z)$ is the ABCD matrix for the optical system between the object and targeted planes.

*Electronic address: khuang@lps.ecnu.edu.cn

†Electronic address: hpzeng@phy.ecnu.edu.cn

Similarly, the pump beam after a digital micromirror device (DMD) is given by

$$E_p(x,y) = E_2(x,y) \times E_P(x,y) \propto e^{-(x^2+y^2)/w_2^2} \times E_P(x,y) \ , \tag{3}$$

where $E_2(x,y)$ is the Gaussian pump laser with a waist of w_2, $E_P(x,y)$ is the modulation pattern. The optical pattern of the pump beam is then transferred into the nonlinear crystal to perform the SFG. The resulting pump spatial distribution is expressed as $E'_p(x,y,z) = \mathcal{F}[E_p(x,y), \mathcal{M}_p(z)]$, where $\mathcal{M}_p(z)$ is the ABCD matrix for the optical system between the DMD and the targeted plane.

The nonlinear interaction between the signal and pump beams can be described by the coupling-wave equations [1]. Under the approximations of paraxial interaction and slowly varying envelope, the SFG field E_{up} can be deduced as

$$\frac{\partial E_{\mathrm{up}}(x,y,z)}{\partial z} = \frac{i2\omega_{\mathrm{up}}^2 d_{\mathrm{eff}}}{k_{\mathrm{up}} c^2} \times E'_s(x,y,z) E'_p(x,y,z) e^{-i\Delta k_z z} + \frac{i}{2k_{\mathrm{up}}} \Delta E_{\mathrm{up}}(x,y,z) \ , \tag{4}$$

where d_{eff} denotes the effective nonlinear coefficient, c is the speed of light in vacuum, z is the propagation direction, and Δk_z represents the longitudinal phase mismatch of the nonlinear conversion process. The involved angular frequencies for the signal, pump and SFG fields satisfy the energy conservation as $\omega_{\mathrm{up}} = \omega_{\mathrm{s}} + \omega_{\mathrm{p}}$.

At the conditions of optimal phase matching $\Delta k_z = 0$ and plane-wave interactions, the SFG intensity after a thin crystal is simply given by

$$I_{\mathrm{up}}(x,y) \propto I_s(x,y) \times I_p(x,y) \ , \tag{5}$$

where I_s and I_p are the intensity distributions for signal and pump electric fields, respectively. Therefore, the detected power of the SFG light is given by

$$P_{\mathrm{up}} = \iint I_s(x,y) \times I_p(x,y) \ dxdy \ . \tag{6}$$

The above equation indicates that solely manipulating the signal beam in the spatial domain is equivalent to spatially modulating the pump beam in the same manner but leaving the signal field unchanged. In other words, the pump pattern can provide an all-optical mask on the signal beam based on the nonlinear wave-mixing operation. Moreover, the mask filtered field is spectrally converted in the visible band, which favors for the sensitive intensity measurement. To perform the single-pixel imaging, a complete set of time-varying pump patterns $I_p^{(\mathrm{m})}(x,y)$ are projected into the nonlinear crystal to get a sequence of intensity values $P_{\mathrm{up}}^{(m)}$. Finally, the two-dimensional object image $O(x,y)$ can simply be reconstructed by

$$O(x,y) = \frac{1}{M} \sum_{m=1}^{M} P_{\mathrm{up}}^{(\mathrm{m})} I_p^{(\mathrm{m})}(x,y) \ . \tag{7}$$

Note that more advanced algorithms can be used to improve the single-pixel imaging performance, as presented in Supplementary Note 5. To include the cutting-off effect for the spatial frequencies in the upconversion imaging, an artificial soft aperture with the Gaussian gradient is added in the relay imaging system for the object image scaling. The size of the aperture is defined by the angular acceptance for the nonlinear crystal [2].

Supplementary Note 2: Experimental setup

The detailed schematic for the experimental setup is illustrated in Supplementary Figure 1. The involved light source stems from a home-made synchronized fiber laser system, which consists of two Yb-doped and Er-doped fiber lasers (YDFL and EDFL). The two fiber lasers are constructed in a polarization-maintaining configuration, and are mode-locked at a repetition rate around 14.6 MHz. The synchronous pulse trains are realized by using the technique of all-optical passive synchronization, such that the relative repetition rate of the two lasers could be stabilized with a high timing precision and a long-term robustness. More information about the synchronized laser system can be referenced to our previous work [3].

The YDFL output is divided into two branches. One portion is sent into a single-mode Yb-doped fiber amplifier (YDFA1). The amplified beam is then spatially combined with the output from the EDFL through a wavelength division multiplexer (WDM). The mixed beam is focused into a periodically poled lithium niobate (PPLN1) crystal to perform the difference-frequency

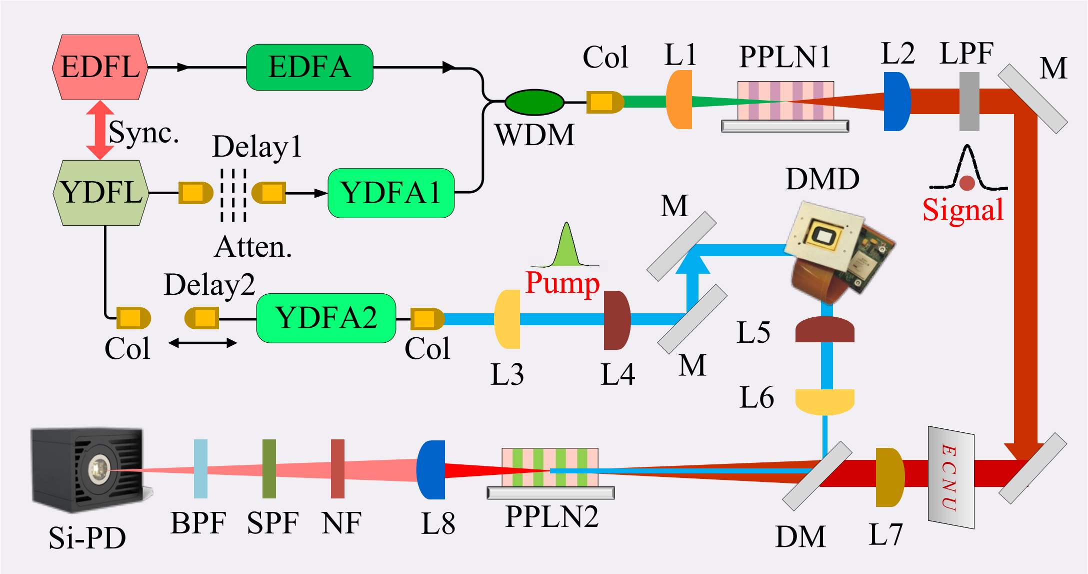

Supplementary Figure 1: Detailed schematic for the experimental setup. MIR signal source at 3070 nm is prepared by the difference frequency generation between two synchronized fiber lasers. The generated MIR signal is illuminated onto a transmission mask, and the formed object image is transferred by a lens into a nonlinear crystal. A structured pump beam at 1030 nm is prepared by a spatial mode modulator based on a DMD. The resulting optical pattern is steered into the crystal for performing the nonlinear spatial modulation based on SFG. The resulting upconverted signal thus carries the spatial information for each projected pattern. The intensity measurement is conducted by a silicon-based sensitive photodiode. The combined knowledges of the acquired intensity values and the predefined patterns enable us to reconstruct the object image with suitable algorithms. EDFL & YDFL: erbium- and ytterbium-doped fiber laser; EDFA: Er-doped fiber amplifier; YDFA: Yb-doped fiber amplifier; WDM: wavelength division multiplexer; Col: collimator; L: lens; Atten: neutral density attenuator; PPLN: periodically poled lithium niobate crystal; M: silver mirror; DM: dichroic mirror; NF: notch filter; LPF, SPF and BPF: long-, short-, and band-pass filter; DMD: digital micromirror device; Si-PD: silicon-based photodiode detector.

generation (DFG), which enables us to prepare MIR pulses at 3070 nm. The poling period of the nonlinear crystal is chosen to be 30.3 μm, and the operation temperature was set to be 40.7 °C to fulfill the quasi-phase-matching condition. Additionally, the DFG conversion efficiency is optimized by carefully tuning the temporal overlap of the two interacting pulses via a delay line (Delay1). A group of calibrated neutral density filters are inserted to control the photon number of the MIR pulse. More details about the MIR generation and power calibration has be presented in Ref. [4]. The other branch of the YDFL is amplified by another amplifier (YDFA2) to boost the average power for serving the pump source. Note that the pump at 1030 nm and the MIR signal at 3070 nm are temporally synchronized, which facilitates to implement the subsequent coincidence-pumping frequency upconversion detection [5].

The signal and pump sources are combined by a dichroic mirror (DM) before being steered into another nonlinear crystal (PPLN2) to perform the SFG. The temporal overlap is adjusted by using another fiber delay line (Delay2), where one of the collimators is placed on a linear translation stage (Thorlabs, LTS300/M). The length of the nonlinear crystal is 10 mm, and the poling period is chosen to be 20.9 μm. The operation temperature is stabilized at 48.6 °C to approach the phase-matching condition. To suppress the background noises, the upconverted light at 771 nm passes through a series of spectral filters. The total transmission is about 80%, and the rejection ratio at the pump wavelength is estimated to be 271 dB. The high-performance filtering system is essential to achieve the single-photon detection sensitivity.

In the experiment, the pump power impinging onto the digital micromirror device (DMD) is set to be about 280 mW, which is below the damage threshold of the spatial modulator. The corresponding conversion efficiency is measured to be about 1% by calculating the photon-flux ratio between the input infrared light and the upconverted SFG light. The modest efficiency is sufficient for us to demonstrate the single-photon imaging performance thanks to the extremely low background noise. Further improvement on the conversion efficiency is possible by boosting the pump power in combination with a high-efficiency spatial modulator. Here, the pulse duration for the pump laser is about 30 ps, which is much longer than that in our previous work [5]. The reduced peak intensity of the pump pulse leads to the decrease of the conversion efficiency. Further improvement on the conversion efficiency can resort to the use of femtosecond pump pulses.

To perform the MIR single-pixel upconversion imaging, the pump beam is spatially modulated by a digital micromirror device (DMD). The spatial modulator (Texas Instrument, DLP650LNIR) contains 1280×800 micromirrors with a 10.8-μm pitch. A dynamic digital illumination with pixel-accurate control is permitted at a binary pattern rate up to 10,752 Hz. The pump beam is enlarged by a beam expander to cover the central pixels of the DMD, and then scaled down to a diameter about 700 μm before being projected into the nonlinear crystal. Meanwhile, the object image under the MIR illumination is transferred by a lens into an intermediated plane at the crystal center, which is spatially modulated by the structured pump field. Through the SFG process, the masked image information is spectrally translated in the visible band, where sensitive silicon-based detectors can be used to register the intensity for each pattern. Two types of detectors are employed depending on the MIR illumination power. A free-space amplified photodetector (Thorlabs, PDA100A2) is used for the sake of convenience in optimizing the imaging performance.

In the case of low-photon-flux illumination, a single-photon counter (Excelitas, SPCM-AQRH-54) based on an avalanche photodiode is used, which is specified with a high detection efficiency of 63% at 771 nm and a low dark noise below 100 Hz. The relevant timing sequence and data acquisition are assisted by a digital controlling unit based on a field programmable gate array (FPGA).

Supplementary Note 3: Operating configurations

The single-pixel imaging relies on the time-varying intensity measurement for a sequence of prior-determined illumination patterns. In our experiment, the involved timing control and data acquisition are realized with a digital controlling unit based on a field programmable gate array FPGA (Altera, Cyclone II). The FPGA chip is specified to have more than 68,0000 logic elements with a high-speed operation up to 250 MHz, which can be programed to provide fast digital inputs and outputs with a high time resolution. As shown in Supplementary Figure 2, the operation period for each pattern T_{loop} is determined by the square wave from the FPGA. The rising edge of the waveform is used to trigger the DMD for pattern switching. For the DMD used in the experiment, the loading process t_1 takes about 50 μs, the reset time t_3 and the waiting time t_4 are set to be 100 and 10 μs, respectively. In the case of using an analogue photodiode as the bucket detector, an illumination time t_2 of 40-μs is used for each pattern, which results in a period

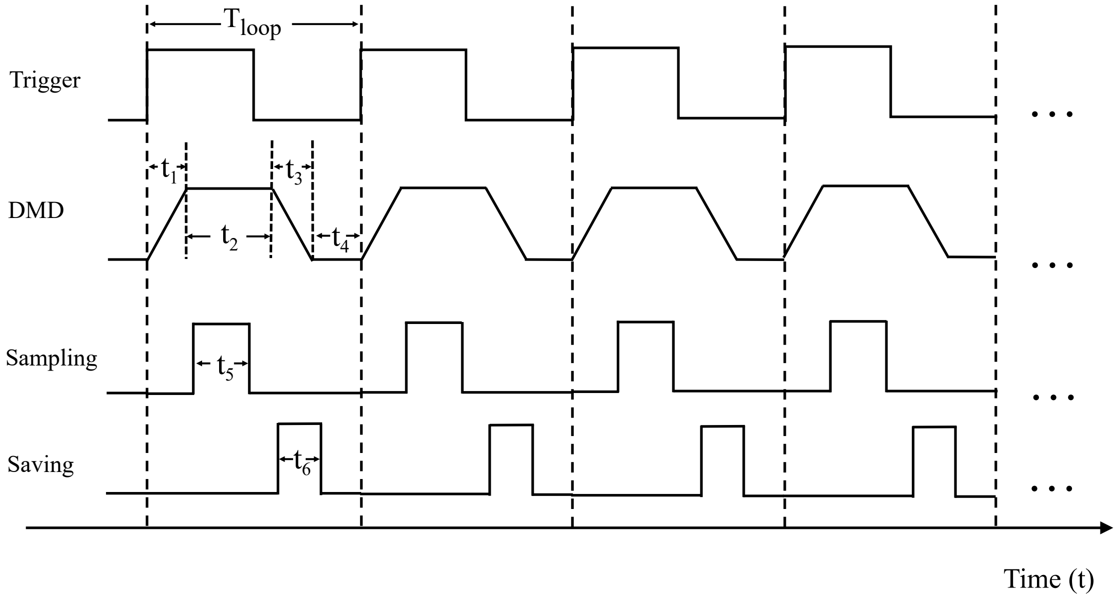

Supplementary Figure 2: Time sequences for the single-pixel imaging operation. The square-wave trigger signal generated from the FPGA is used to synchronize the related steps of pattern illumination, intensity measurement, and data storage. Note that a small rising time t_1 is needed to reach a stable pattern illumination with a period of t_2. Similarly, a trailing time of t_3 is needed to completely reset the DMD. t_4 is the waiting time between sequential patterns. $t_{5,6}$ denote the sampling time for the intensity measurement and the saving time for the data storage, respectively. The whole package of data will be transferred to a computer after one complete round of pattern playing.

T_{loop} of 200 μs, corresponding to a refreshing rate of 5 kHz. It is worth noting that the picture time T_{loop} is optimized to obtain stable optical patterns, which can be further reduced by using a more advanced DMD. In the experiment, a 12-bit analog-to-digital converter (Analog Devices, AD7091R8) is used to sample the input voltage at a throughput rate of 1 MHz.

In the low-light-level regime, a single-photon counting module (SPCM) is used for the single-pixel detection. The digital detector will output a TTL pulse when recording a photon. A frequency counter is implemented by the FPGA, which can precisely count the number of the rising edge due to the high-speed sampling clock. In order to accumulate enough photons for the subsequent image reconstruction, a displaying time for each pattern is typically set to be from 5 ms to 1 s depending on the illumination power.

The measurement result for each pattern is quickly stored into the embedded random access memory (RAM). Once the whole set of patterns are played, all the temporally saved data is transferred into a computer. A first-in-first-out (FIFO) buffer architecture is used for the data transferring. In this configuration, the data first written into the buffer comes out of it first, which makes sure that the transferred values correctly correspond to the defined pattern order. A graphic user interface (GUI) based on LabView is developed to set the relevant parameters and display the reconstructed images, which facilitates the experimental optimization and characterization of the single-pixel imaging system.

Supplementary Note 4: Nonlinear structured modulation

The proposed approach based on the nonlinear structured detection is the core to realize the sensitive MIR single-pixel imaging at the single-photon level. In this modality, the required masks for the MIR radiation are realized by an optically-controlled spatial modulation within a nonlinear crystal. Consequently, the pump structured patterns are mapped onto the targeted MIR object image through the SFG process. Simultaneously, the screened MIR spatial information is spectrally translated into the upconverted beam at the visible band. The spatial mapping capability has been illustrated in Supplementary Figure 3. We load three exemplary Hadamard patterns into the DMD, and the generated intensity distributions of the pump at the nonlinear crystal are shown in Supplementary Figures 3(b1-b3). The brighter pixels at the central part are ascribed to the inhomogeneous Gaussian illumination in the experiment. Supplementary Figures 3(c1-c3) show the corrected intensity patterns, which are closed to the theoretical Hadamard masks.

In contrast to previous configuration of optically controlled modulators [6–8], the unique feature for the proposed nonlinear spatial modulation is the ability to spectrally convert the masked field into a replica at a disparate wavelength. To illustrate this point, we remove the object in the MIR signal path, and inject the MIR beam into the crystal. In this case, the pump pattern will be imprinted directly onto the upconverted beam. The resulting upconverted patterns and the corrected ones are illustrated in Supplementary Figures 3(d1-d3, e1-e3), which are expected to resemble the Hadamard matrices. The involved high-fidelity spatial mapping lays the foundation to implement the MIR single-pixel imaging based on the nonlinear structured detection.

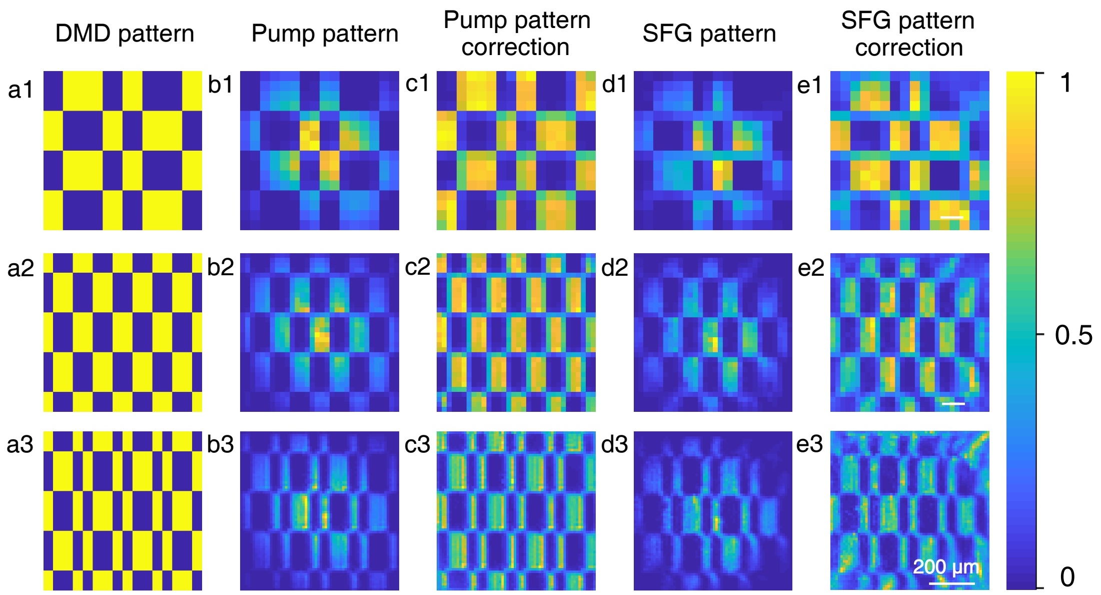

Supplementary Figure 3: Optically-controlled spatial modulation via the structured pump illumination. (a1-a3) Three representative Hadamard patterns loaded into the DMD. (b1-b3) Prepared optical patterns of the pump beam at the center of the nonlinear crystal. (c1-c3) Corrected pump patterns by taking into account the inhomogeneous Gaussian beam profile in the experiment. (d1-d3) Generated upconverted patterns without inserting an object in the MIR illumination path. (e1-e3) Corrected SFG patterns for the Gaussian intensity distribution.

Supplementary Note 5: Reconstruction algorithms

A single-pixel camera requires the use of a series of masks to acquire the spatial information with a bucket detector. Fundamentally, a type of polymorphic scanning is performed with a single pixel to weakly sense the intensity information from many spatial points at once [9]. Therefore, specific algorithms are usually needed to reconstruct the object image based on the measured intensities and predefined patterns. Practically, a careful design for a set of masks plays an important role in improving the single-pixel imaging performance [10]. In our experiment, we have adopted three types of encoding masks to evaluate the proposed MIR single-pixel imaging approach based on the nonlinear structured detection.

The most straightforward way to obtain an image is to illuminate each pixel sequentially, effectively raster scanning a single pixel over the scene. This per-pixel measurement works well with high light levels due to the sufficient signal-to-noise ratio (SNR), as shown in Supplementary Figure 4(a1). However, the raster scanning is inefficient to use the available light, and thus usually suffers from the detector noise in the case of small signals emanating from a single aperture. This issue is more prominent at the low-light-level illumination or with a smaller aperture size for increased number of pixels. As shown in Supplementary Figure 4(a3), the object can barely been recognized due to the low SNR in the case of 64×64 pixels.

In comparison, multiaperture masking schemes offer the advantage of minimizing the effect of detector noise by using more light in each measurement. To this end, a DMD is used as a spatial

light modulator to generate binary matrices for illumination. We first test the random encoding, which is commonly used in classical ghost imaging where a set of speckle patterns is prepared by a pseudo-thermal light field. In this scenario, M random patterns $P(x, y)$ are used to construct an object image $O(x, y)$. The measured intensity for i^{th} pattern denotes as S_i. Consequently, the object image can be estimated by [11]:

$$O(x, y) = \sum_{i=1}^{M} (P_i(x, y) - \langle P(x, y) \rangle) \times (S_i - \langle S \rangle) \ , \tag{8}$$

where $\langle\rangle$ denotes the average value. The experimentally reconstructed images are shown in Supplementary Figures 4(b1-b3) for the number of pixels form 16×16, 32×32 and 64×64, respectively. In comparison to the results based on raster scanning, the use of random patterns is indeed beneficial to improve the SNR especially for a smaller pixel aperture.

Another effective multipixel masking approach can resort to Hadamard patterns. We consider the construction of an N-pixel image, which can be represented by a flattened vector $O_{N\times1}$ with N elements. Similarly, each masking pattern can be flatten into a row, thus resulting in a matrix $P_{N\times N}$ for including N patterns. Consequently, the measurement results can be represented by a vector $S_{N\times1}$ mathematically expressed as

$$S_{N\times1} = P_{N\times N} \cdot O_{N\times1} \ . \tag{9}$$

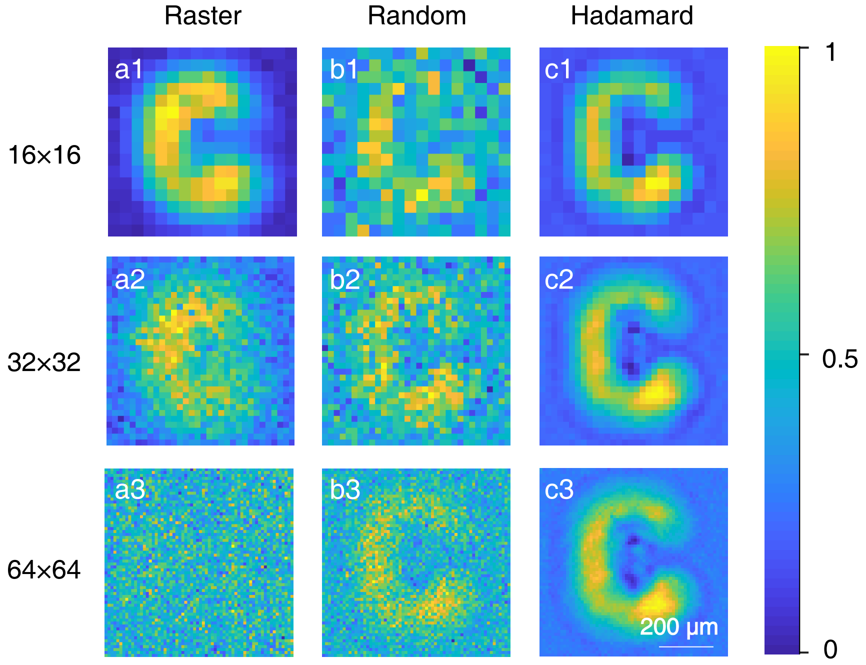

Supplementary Figure 4: Imaging performance comparison among raster, random and Hadamard encodings. (a1-a3) Images obtained using raster masks with increasing number of pixels from 16×16, 32×32 and 64×64, respectively. (b1-b3, c1-c3) Reconstructed images with multipixel masks based on Radom (b1-b3) and Hadamard (c1-c3) patterns with increasing number of pixels from 16×16, 32×32 and 64×64, respectively. Note that all the images are acquired at the same MIR illumination power.

Therefore, the image vector can be obtained through matrix inversion $O = P^{-1} \cdot S$. Note that the resulting vector should be reformatted into a two-dimensional matrix for properly displaying the object image. Intriguingly, the Hadamard matrix is orthonormal. This feature ensures that the scalar product between any two distinct rows is null, that is, each row is orthogonal to every other one. The inverse of the Hadamard matrix can simply be obtained via the transpose operation, which allows us to perform a fast image reconstruction. The corresponding results are presented in Supplementary Figures 4(c1-c3), which indicate a superior performance for various pixel numbers. It has been proved that Hadamard matrices constitute a set of bases that minimize the mean squared error for each pixel in the image [12]. Additionally, differential intensity measurement is performed in the experiment to reduce the low-frequency source oscillations [6], where the pattern and its photographic negative are displayed in a successive manner.

Furthermore, the MIR single-pixel imaging is investigated by adopting the compressive sensing technique to reconstruct the image by undersampling the object [13]. The smaller number of measurements favors significant reduction of the acquisition time. In the case of M measurements smaller than the pixel number of N, Eq. (9) becomes

$$S_{M\times 1} = P_{M\times N} \cdot O_{N\times 1} = P_{M\times N} \cdot \Psi_{N\times N} \cdot X_{N\times 1} = \Theta_{M\times N} \cdot X_{N\times 1} \ , \tag{10}$$

where the object image is expanded in a sparse representation by $O_{N\times 1} = \Psi_{N\times N} \cdot X_{N\times 1}$. The target image vector is called K-sparse if there are K nonzero elements in $X_{N\times 1}$. The so-called restricted isometry property (RIP) for the matrix $\Theta_{M\times N}$ is a sufficient condition for a stable inverse for both K-sparse and compressible signals [14]. In our experiment, we take a set of random patterns to construct the measurement matrix $P_{M\times N}$, and recover the image by using an optimization algorithm to minimize the l_1 norm:

$$\widehat{X} = \text{argmin}\|X'\|_1 \qquad \text{such that} \quad \Theta \cdot X' = S \ . \tag{11}$$

This is a convex optimization problem that conveniently reduces to a linear program known as basis pursuit [14]. Specifically, we use a primal-dual algorithm for the linear programming. The reconstruction code is modified from l1-MAGIC that is a collection of MATLAB routines for solving the convex optimization programs [15].

Finally, the machine learning approach is introduced to our MIR single-pixel imaging system, where deep learning algorithms based convolutional neural networks (CNNs) are used to perform the image reconstruction. The deep learning technique allows to adapt the sampling basis to be most efficient to sample a scene, thus leading to the image recovery with a minimal number of measurements. Moreover, the CNN can be used to suppress the image noises by training a highly flexible and effective deep denoiser. In our experiment, we adopt the so-called DRUNet (Dilated-Residual U-Net) to reconstruct images at low-light-level scenarios. The DRUNet approach is featured with the ability to capture both the local and contextual information, which can significantly suppress the noises [16]. The implemented code is adapted from the one developed by K. Zhang *et al.,* [17], which enables us to achieve a superior Gaussian denoising performance.

Supplementary Note 6: Photon-sparse MIR single-pixel imaging

The nonlinear structured detection facilitates the simultaneous operations of spatial mapping and spectral transferring, which enables us to demonstrate the MIR single-pixel imaging at the single-photon level. In the photon-sparse regime, the integration time for the single-photon detector is essential to obtain a stable photon-counting rate, which plays an important role in optimizing the quality of the reconstructed image. The imaging performances at various periods in frame displaying are illustrated in Supplementary Figures 5(a-e, f-j, k-o) for the pixel numbers of 16×16, 32×32, and 64×64, respectively. Note that the illumination power is set to be 1 photon/pulse in the experiment. Generally, a longer exposure time for each pattern helps to obtain a better image contrast, which is expected due to the improved signal-to-noise ratio of the intensity measurement. Indeed, in the single-photon regime, photon-counting fluctuations become prominent due to the intrinsic Poissonian photon statistics for the coherent-state light. The ratio between the standard deviation and the mean value for the recorded photon counts is inversely proportional to the square root of the photon number within the integration time. Consequently, a better photon-counting stability can be achieved with a longer integration time. It is also worth noting that the imaging quality for larger pixel numbers is more sensitive to the integration time as the photons distributed into an individual pixel become less, which is manifested in Supplementary Figures 5(k-o).

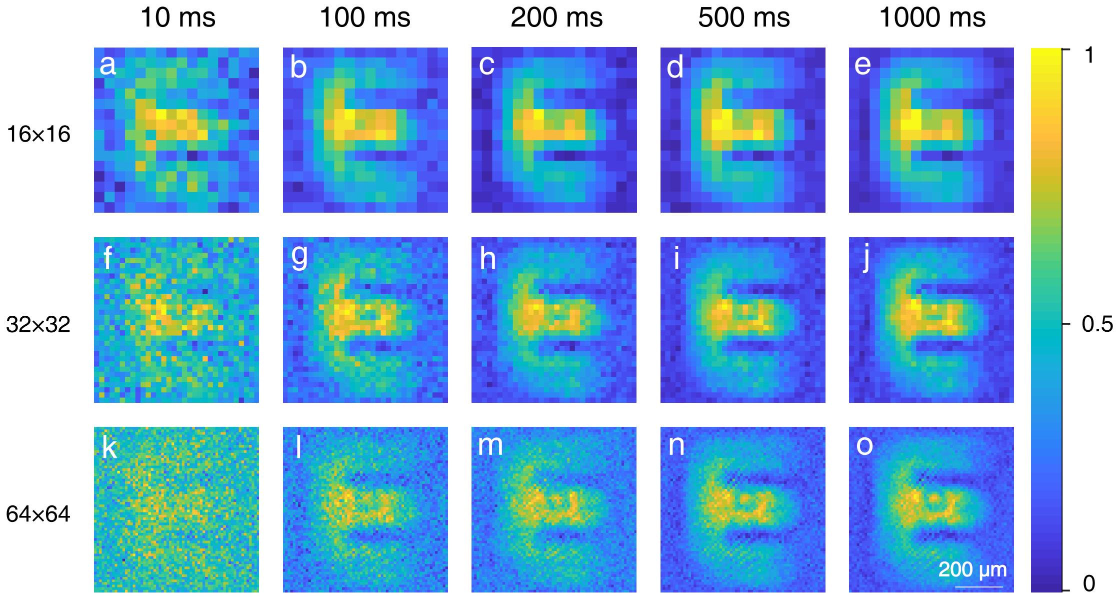

Supplementary Figure 5: Single-pixel imaging performance as increasing the displaying time for each pattern from 10 ms to 1 s. The imaging quality is also investigated for reconstructed pixels of 16×16 (a-e), 32×32 (f-j), and 64×64 (k-o), respectively. Note that the illumination power is kept at 1 photon/pulse.

Supplementary Note 7: Proof-of-principle imaging through a silicon wafer

The MIR spectrum is pertinent to the transparent window for silicon and germanium materials, which renders the MIR imaging useful in non-destructive defect inspection for semiconductor chips. Here, we perform a proof-of-principle demonstration for the transmission imaging through a silicon wafer. The wafer has a thickness of 200 μm and is polished at the two surfaces. As shown in Supplementary Figure 6(a), a five-pointed star is printed by using the laser etching technique onto the surface of a silicon substrate. Specifically, a high-power laser (JPT Electronic, YDFLP-20-M6) at 1064 nm is used as the sculpting light, which can deliver a maximum average power of 20 W. In the preparation of the sample, the pulse parameters are optimized with a pulse duration of 10 ns, a repetition rate of 250 kHz, and a pulse energy of 32 μJ. The speed of the laser writing is controlled to be 5 mm/s.

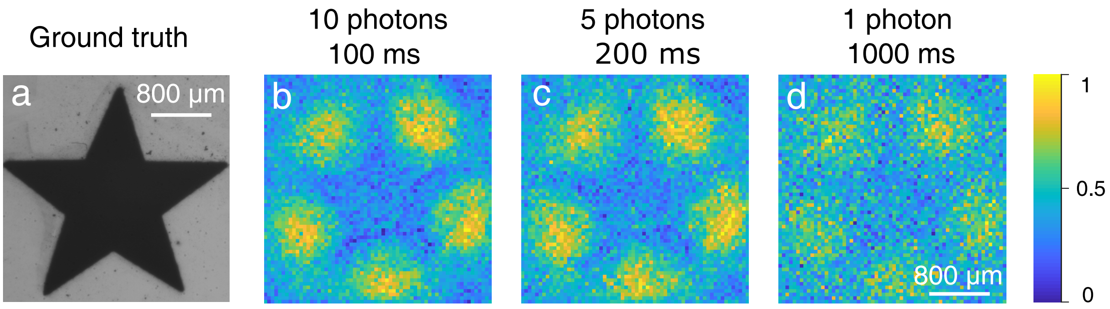

Supplementary Figure 6: Single-pixel imaging at the photon-starving regime through a silicon wafer engraved with a five-pointed star. (a) Object image. (b-d) Reconstructed images under low-light-level illuminations with 10, 5 and 1 photons/pulse, respectively.

There will be stronger scattering at the ablated part, thus leading to a reduced power transmission. The transmitted infrared light generates the object image, which is then mapped into the nonlinear crystal to perform the MIR single-pixel imaging based on the nonlinear structured detection. Supplementary Figure 6(b) presents the sensitive MIR imaging for an illumination power of 10 photons/pulse. The integration time is set to be 100 ms for recording sufficient photons by the silicon bucket photon counter. As shown in Supplementary Figure 6(d), the imprinted pattern can still be identified as decreasing the illumination power down to 1 photon/pulse, albeit with a longer integration time for the photon-counting measurement. Our ongoing work will focus on the improvement of the field of view and spatial resolution for the MIR single-pixel imaging system, which would provide a useful tool in interior structure examination for semiconductor devices and label-free diagnosis for biochemical samples.

---

[1] Boyd, R. W. *Nonlinear Optics* (4th Edition, Elsevier, 2020).

[2] Barh, A., Rodrigo, P. J., Meng, L., Pedersen, C. & Tidemand-Lichtenberg, P. Parametric upconversion imaging and its applications. *Adv. Opt. Photon.* **11**, 952-1019 (2019).

[3] Zeng, J., Li, B., Hao, Q., Yan, M., Huang, K. & Zeng, H. Passively synchronized dual-color mode-locked fiber lasers based on nonlinear amplifying loop mirrors. *Opt. Lett.* **44**, 5061-5064 (2019).

[4] Huang, K., Wang, Y., Fang, J., Kang, W., Sun, Y., Liang, Y., Hao, Q., Yan, M. & Zeng, H. Mid-infrared photon counting and resolving via efficient frequency upconversion. *Photon. Res.* **9**, 259-265 (2021).

[5] Huang, K, Fang J., Yan M., Wu E. & Zeng H. Wide-field mid-infrared single-photon upconversion imaging. *Nat Commun.* **13**, 1077 (2022).

[6] Stantchev, R. I., Sun, B., Hornett, S. M., Hobson, P. A., Gibson, G. M., Padgett, M. J. & Hendry, E. Noninvasive, near-field terahertz imaging of hidden objects using a single-pixel detector. *Sci Adv.* **2**, e1600190 (2016).

[7] Stantchev, R. I., Yu, X., Blu, T. & Pickwell-MacPherson, E. Real-time terahertz imaging with a single-pixel detector. *Nat. Commun.* **11**, 2535 (2020).

[8] Zhao, J., E, Y., Williams, K., Zhang, X.-C. & Boyd, R. W. Spatial sampling of terahertz fields with sub-wavelength accuracy via probe-beam encoding. *Light Sci. Appl.* **8**, 55 (2019).

[9] Edgar, M. P., Gibson, G. M. & Padgett, M. J. Principles and prospects for single-pixel imaging. *Nature Photon.* **13**, 13-20 (2019).

[10] Gibson, G. M., Johnson, S. D. & Padgett, M. J. Single-pixel imaging 12 years on: a review. *Opt. Express.* **28**, 28190-28208 (2020).

[11] Moreau, P. A., Toninelli, E., Gregory, T. & Padgett, M. J. Ghost imaging using optical correlations. *Laser Photon. Rev.* **12**, 1700143 (2017).

[12] Harwit, M., & Sloane, N. J. A. Hadamard Transform Optics (Academic Press, New York, 1979).

[13] Eldar, Y. C. & Kutyniok, G. Compressed Sensing: Theory and Applications (Cambridge Univ. Press, Cambridge, 2012).

[14] Baraniuk, R. G. Compressive sensing. *IEEE Signal Process. Mag.* **24**, 118-121 (2007).

[15] Candès E. & Romberg, J. $l1$-magic: recovery of sparse signals via convex programming. https://candes.su.domains/software/l1magic/downloads/l1magic.pdf .

[16] Zhang, K., Li, Y., Zuo, W., Zhang, L., Gool, L. V. & Timofte, R. Plug-and-play image restoration with deep denoiser prior. *IEEE. Trans. Pattern Anal. Mach. Intell.* 3088914 (2021).

[17] https://github.com/cszn/DPIR.
