# Supplementary material for: Mid-infrared single-pixel imaging at the single-photon level
Source: Nat Commun. 2023 Feb 25;14:1073. doi: 10.1038/s41467-023-36815-3 (PMC9968282; doi:10.1038/s41467-023-36815-3)
Supplement: Supplementary file 3 — Description of Additional Supplementary Files [file 41467_2023_36815_MOESM3_ESM.pdf]

Description of Additional Supplementary Files

Supplementary Movie 1: Nonlinear structured modulation of optical pump patterns for a full set of Hadamard matrices. The images at the first column are the patterns loaded into the digital micromirror device. The images at the second column correspond to the recorded intensity patterns for the pump beam within the nonlinear crystal. The intensity patterns corrected for the Gaussian pump profile are given at the third column. The pixel number is set to be 16×16.

Supplementary Movie 2: Mid-infrared single-pixel imaging at real time. The left and right videos are recorded for a moving object with reconstructed pixels of 16×16 and 32×32, respectively. The frame rates for the two settings are about 10 fps and 2.5 fps. Note that these videos are recorded in real time, which include the time for the data acquisition and the image reconstruction.
